# *Beauveria felina* Accelerates Growth When Competing With Other Potential Endophytes

**DOI:** 10.1111/1758-2229.70067

**Published:** 2025-01-16

**Authors:** Wojciech Pijanowski, Sebastian Chmielewski, Wojciech Wysoczański, Polina Havrysh, Katarzyna Turnau, Marlena Lembicz

**Affiliations:** ^1^ Department of Systematic and Environmental Botany Faculty of Biology, Adam Mickiewicz University Poznań Poland; ^2^ Evolutionary Biology Group, Faculty of Biology, Adam Mickiewicz University Poznań Poland; ^3^ Institute of Plant Genetics, Polish Academy of Sciences Poznań Poland; ^4^ Institute of Environmental Sciences Jagiellonian University in Kraków Kraków Poland

**Keywords:** biocontrol agent, biotic effects, endophytic fungi, growth model, mycelium network

## Abstract

The fungus *Beauveria felina* is often classified as one of the so‐called good biocontrol agents. However, no information is available about the growth of this entomopathogenic fungus in the presence of other endophytic fungi, which are usually found in plant tissues. Effects of fungal interactions vary from inhibiting the activity of a biocontrol agent to stimulating its effect on the targeted pathogen. This study compared the growth rate of *Beauveria felina* alone and in interaction with other endophytic fungi. In the presence of each competitor (*Gliomastix polychroma* or *Rhodotorula mucilaginosa* ), *B. felina* grew faster than in the control. In the interaction between *Beauveria felina* and *Gliomastix polychroma*, an inhibition zone was formed between their mycelia. This is the first report showing the response of its mycelium to biotic stress caused by the presence of other fungi.

## Graphical abstract

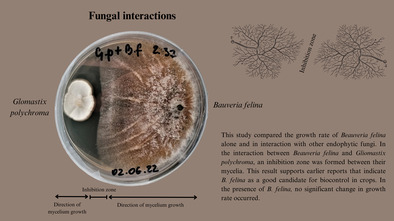

## 1 Introduction

Currently, scientists search intensively for organisms for biocontrol agents of crop pests. Biocontrol agents selectively protect important organisms in an environment‐friendly way (Backman and Sikora 2008; Thambugala et al. 2020). Biocontrol agents are alternatives to pesticides, antibiotics and fungicides. Chemical pest control harms organisms that perform ecosystem services and can benefit the crop. Thus, crop protection should take into account the biodiversity of the whole environment where the crop is grown.

The endosymbionts forming the plant microbiome, including endophytic fungi, are a group of organisms that include potential biocontrol agents (Baiyee et al. 2019; Segaran and Sathiavelu 2019; Agrawal et al. 2021; Adeleke et al. 2022; Sirikamonsathien, Kenji, and Dethoup 2023). Over 20 years of intensive basic research on fungal endophytes has provided extensive knowledge about their species diversity as well as biological, chemical and ecological activity (Cheplick and Faeth 2009; Rodriguez et al. 2009; White and Bacon 2012; Alam et al. 2021). Their chemical activity mostly involves production of various metabolites (Vázquez de Aldana et al. 2010; Zhang et al. 2012; Saikkonen, Gundel, and Helander 2013). Nearly every fungal species produces a different, specific range of chemical compounds. In turn, metabolites affect the host plants of fungal endophytes, especially their growth, reproduction and self‐defence against pathogens and other stress factors (Latz et al. 2018). However, the effects of the interactions vary depending on genotype (of both the plant and the fungus), environmental conditions and when the interaction started.

In the search for fungi for biocontrol, scientists pay special attention to endophytic fungal species that limit the abundance of pathogens and thus can become natural, ‘living’ biopesticide, biofungicide or bioinsecticide. The market already has many commercial products containing microbial biological control agents (BCA), which are used in agriculture, for example, products based on *Trichoderma* spp. or *Bacillus thuringiensis* (Menzler‐Hokkanen 2006; Thambugala et al. 2020). A good candidate for a biocontrol agent is an entomopathogenic fungus, *Beauveria felina* (DC.) J. W. Carmich 1980, an ascomycete of the family Cordycipitaceae. Researchers have reported that the fungus produces several secondary metabolites, for example, as many as seven cyclopeptides, including six novel cyclodepsipeptides: isaridin I–N (Jiang et al. 2023). The isaridin K produced by *B. felina* contains a peptide skeleton with N‐methyl‐2‐aminobutyric acid, which is rare in natural cyclopeptides. The biological tests performed so far have shown that this compound can significantly inhibit mycelium growth of *Geotrichum citri‐aurantii*, which causes a disease of citrus fruits and leads to huge economic losses (Jiang et al. 2023). Moreover, *B. felina* infests an invasive insect species, *Spodoptera frugiperda* , which is a very dangerous pathogen of maize (Ramanujam et al. 2021).

Results of the cited and earlier studies (e.g., Pedras, Zaharia, and Ward 2002; Vázquez et al. 2005; Morais‐Urano, Chagas, and Berlinck 2012; Vega 2018) have demonstrated the potential for practical use of entomopathogenic fungi such as *Beauveria felina* for biocontrol. However, there are no reports concerning its response to the presence of other endophytic fungi, which are usually found in plant tissues. Effects of the fungal interactions vary from inhibiting the action of a biocontrol agent to stimulating its effect on the targeted pathogen. It is difficult to compare costs and profits in the multiple fungal interactions within plants. In this study, we investigated the strength of the influence of the fungal network of *B. felina* on other endophytic fungi, which can be present in plants treated with biocontrol. Data on the growth rate of *B. felina* , and thus its tolerance to the presence of other species that have colonised the plant, are important before applying the metabolites of the biocontrol agent to fight plant pathogens. We tested two hypotheses: (1) the growth of *Gliomastix* is slower in the presence of another endophytic fungus (in our case, *Rhodotorula* and *Beauveria felina*) and (2) it is possible to eliminate *Gliomastix* and *Rhodotorula* by using another endophytic fungus, which could be a BCA if the endophytes negatively affect the host.

This is the first report showing the response of the network of *B. felina* to biotic stress caused by the presence of other fungi.

## 2 Material and Methods

### 2.1 Fungal Species and Their Identification

The growth of the mycelial network of the ascomycete *Beauveria felina* was explored both in an axenic culture and in the presence of other fungi: an Ascomycete *Gliomastix polychroma* J. F. I I. Beyma, Matsushima, of the family Bionectriaceae and a Basidiomycete, *Rhodotorula mucilaginosa* A. Jorg. F. C. Harrison, of the family Sporidiobolaceae. *B. felina* originated from a collection of fungal strains isolated from zinc spoil tips on Sardinia. *G. polychroma* was isolated from an alga, *Klebsormidium flaccidum* Kutzing, S. Mattox et Blackwell, whereas *R. mucilaginosa* was from *Plantago ovata* growing on a copper spoil tip in Sweden (Orebro). All the fungi were identified by molecular methods and are stored at −80°C in a laboratory of the Institute of Environmental Sciences, Jagiellonian University, Krakow, Poland. Fungal identification was based on sequencing of the internal transcribed region (ITS) of rDNA amplified by polymerase chain reaction (PCR). DNA extraction was performed with the EZ‐10 Spin Columns Fungal Genomic DNA Mini‐Preps Kit (BioBasic, Canada). PCR was performed in a 25‐μL reaction mixture containing 10 ng of DNA matrix, 9.5 μL of nuclease‐free water, 12.5 μL of Maxima Hot Start Green PCR Master Mix (Thermo Scientific, Waltham, MA, USA), and 1 μL of each of the primers: ITS1F (Gardes and Bruns 1993) and ITS4 (White, Bruns, and Taylor 1990), at 10 pmol concentration for each sample. PCR conditions included initial denaturation at 95°C for 3 min, 35 cycles of denaturation at 95°C for 30 s, annealing at 55°C for 30 s, elongation at 72°C for 45 s and final elongation at 72°C for 5 min. The presence of PCR products was viewed in 1.5% agarose gels stained with GelRed (Biotium, Hayward, CA, USA). Isopropanol and sodium acetate precipitation were employed to purify the PCR products (Green and Sambrook 2017). The ITS4 primer was used to read the sequences. Nucleotide sequences were analysed using Chromas software (www.technelysium.com.au) and then compared with published sequences in the NCBI database (www.ncbi.nlm.nih.gov) with the use of the BLASTn algorithm. The identification of the fungal species was confirmed if at least 98% of the sequence was similar in the ITS region to the reference sequences deposited in the NCBI database: *Rhodotorula mucilaginosa* accession number: ON259950; *Gliomastix polychroma* accession number: OR607959 and *Beauveria felina* accession number: OR604691.

### 2.2 Fungal Strain Culture and Measurements

The fungi were cultured in vitro on potato dextrose agar (PDA) with an antibiotic, chloramphenicol. Fungal networks of one species (monocultures) incubated for 11 weeks were used as the control. They were compared with dual cultures, that is, Petri dishes with pairs of the studied species, in all three possible combinations, incubated for 5 weeks. Fresh Petri dishes were inoculated with fragments of mycelium, covering about 5 mm^2^ each. Every fungal species in isolation (monoculture) and every interaction (dual culture) were incubated with 50 replicates. Thus, 300 Petri dishes were observed in total: 150 of the control sample and 150 of the test sample (Table 1), although some results were rejected because of bacterial infections. We excluded from further observations of interactions between fungi all the Petri dishes where bacterial infections appeared. The growing fungal networks were scanned with a scale bar every 3 days for 5 weeks using a Zeutschel OS14000A2 scanner. In total, for each of the 150 Petri dishes in the control sample, 26 such mycelium area measurements were taken, whereas in the sample, 13 measurements were taken for each.

**TABLE 1:** Comparison of numbers of cultured and analysed fungal isolates. The differences were due to infections.

Control sample		
Species	Number of cultured isolates	Number of isolates used for data analysis
*Gliomastix polychroma*	50	42
*Rhodotorula mucilaginosa*	50	34
*Beauveria felina*	50	35
Total	150	111

Test sample		
Interacting species	Number of cultured isolates	Number of isolates used for data analysis
*Gliomastix polychroma +* *Rhodotorula mucilaginosa*	50	42
*Gliomastix polychroma + Beauveria felina*	50	40
*Rhodotorula mucilaginosa* *+ Beauveria felina*	50	43
Total	150	125

### 2.3 Statistical Analysis

The surface area of mycelia was individually plotted by time to identify potential outliers. One dish containing *B. felina* and *G. polychroma* was excluded from the analysis due to a short‐term reduction in the mycelial surface area of both species, which indicated possible culture contamination. The growth rate of mycelia was modelled using the GrowthCurver package (Sprouffske and Wagner 2016). After inspection of the data fit to the generated growth curves, we extracted the growth rate parameter (*r*) for each observation (Table 3). Firstly, the growth rate of three species in the control sample (i.e., monocultures) was compared using simple linear models, and pairwise comparisons were made using Tukey's HSD post hoc test. Next, the same methods were employed to investigate if the presence of another species in the same dish affected the growth rate. The growth rate of *R. mucilaginosa* showed heteroscedasticity; therefore, in comparison with this species, we used square‐rooted data. Growth curve analysis was conducted using R statistical software (v. 4.1.3, R core team), and data were visualised with *ggplot2* (Wickham, 2008) and *rstatix* (Kassambara 2023). Tukey's test was performed using a *multcomp* package.

## 3 Results

**FIGURE 1:**
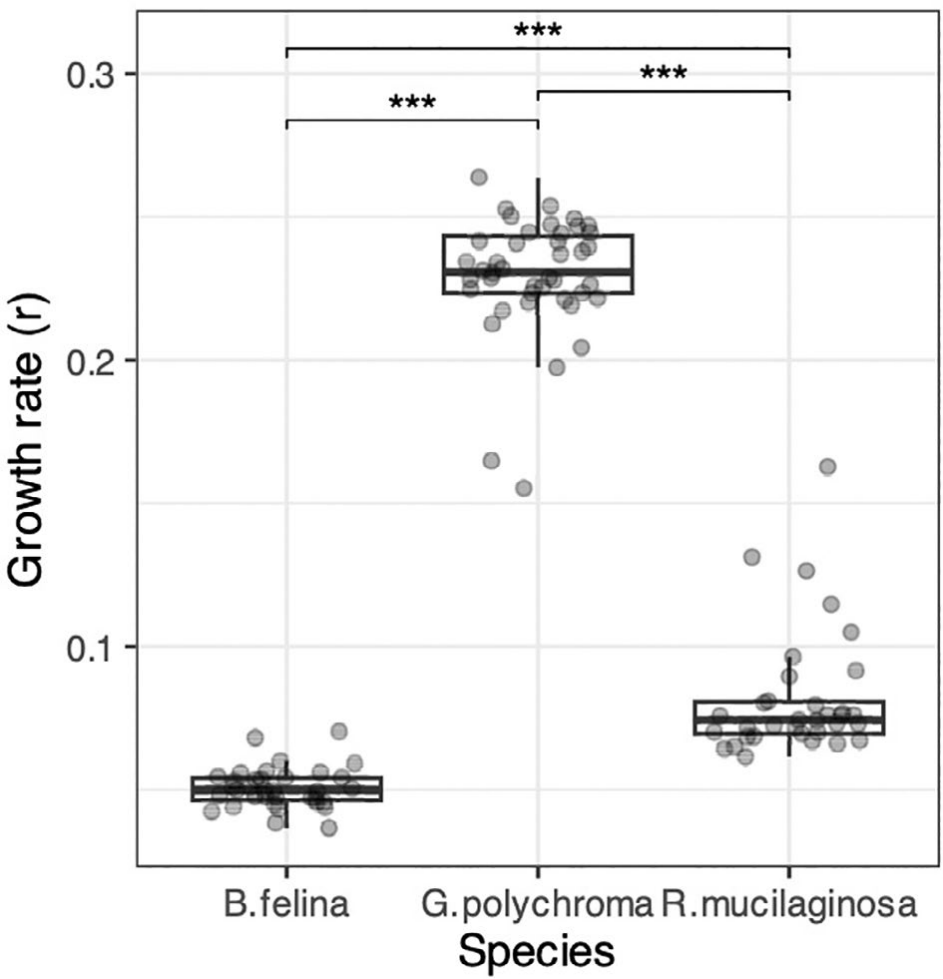
Growth rates of fungal endophytes in vitro in the control treatment. The boxes represent the median and the 25th and 75th percentiles, with whiskers extending to 1.5 times the interquartile range. Circles indicate individual growth rate of each dish. The significance of differences was tested with Tukey's HSD test (***p < 0.0001).

**FIGURE 2:**
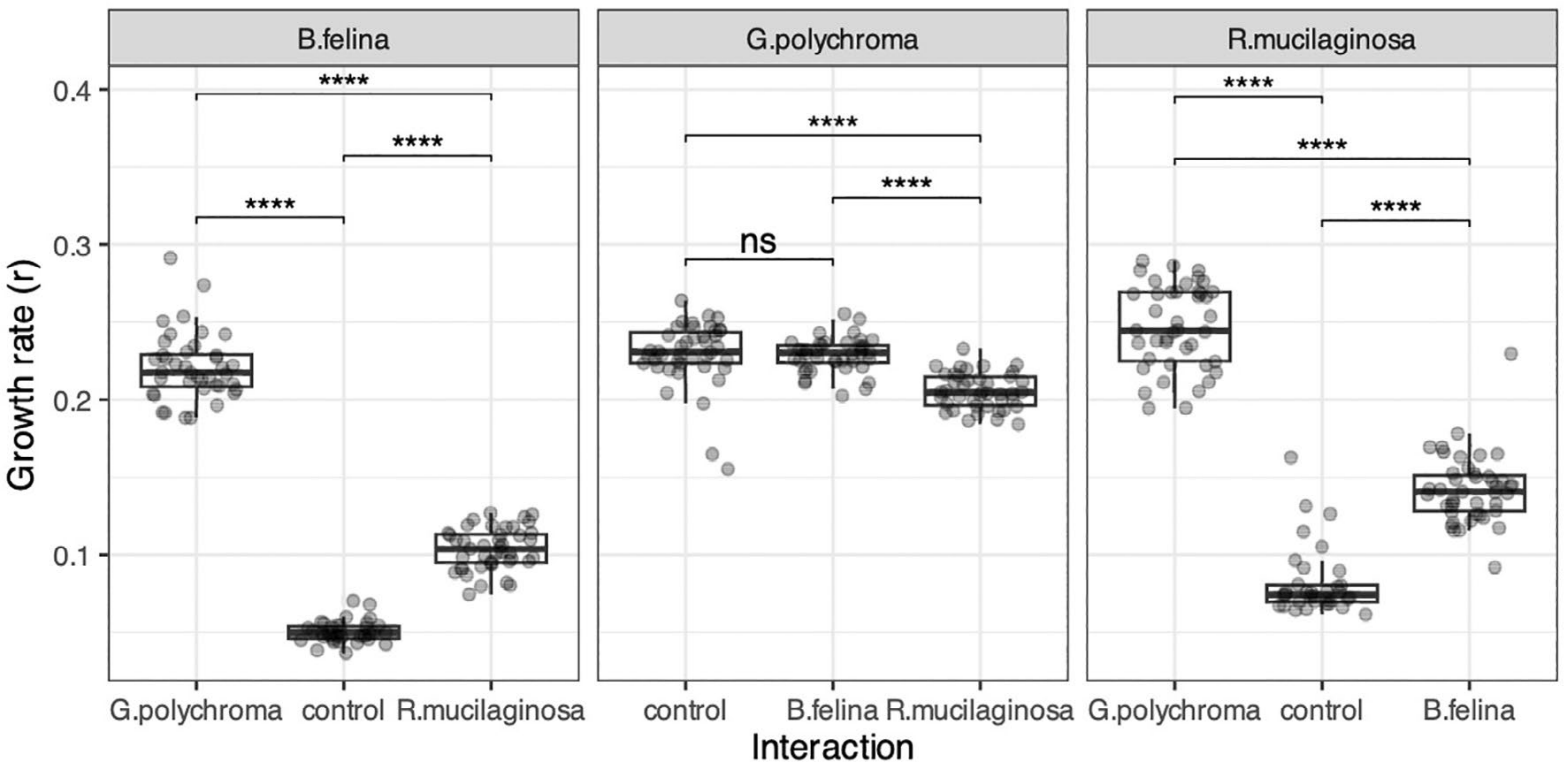
Growth rates of fungal endophytes in vitro in the presence of competitors. The boxes represent the median and the 25th and 75th percentiles, with whiskers extending to 1.5 times the interquartile range. Circles indicate individual growth rate of each dish. Significance was tested with Tukey's HSD test (***p < 0.0001).

The rate of mycelium growth of *Gliomastix polychroma* in the control sample (no competition) was significantly higher than that of *Beauveria felina* (Figure 1, Tukey's HSD test, *p* < 10^−10^) and *Rhodotorula mucilaginosa* (Tukey's HSD test, *p* < 10^−10^). Irrespective of competitor species, its presence significantly increased the growth rate of all the studied species, as compared to the control (Figure 2), except for *G. polychroma*, whose growth rate did not change in the presence of *B. felina* (*p =* 0.957). However, in the presence of *G. polychroma*, the growth rate of *B. felina* increased 4.3‐fold, compared to the control (Figure 2, Table 2; Tukey's HSD test, *p* < 10^−16^, whereas in the presence of *R. mucilaginosa* , it doubled Tukey's HSD test, *p* < 10^−16^).

**TABLE 2:** Summary of growth rates of fungal endophytes in vitro without competitors (control) or in the presence of another species (bicultures).

Species	Interaction	Mean growth rate	Growth rate standard deviation
*Beauveria felina*	Control	0.051	0.007
	*R. mucilaginosa*	0.104	0.013
	*G. polychroma*	0.221	0.022
*Gliomastix polychroma*	Control	0.230	0.021
	*B. felina*	0.229	0.011
	*R. mucilaginosa*	0.205	0.011
*Rhodotorula mucilaginosa*	Control	0.082	0.022
	*B. felina*	0.142	0.022
	*G. polychroma*	0.247	0.027

**TABLE 3:** Growth rate pairwise comparisons using Tukey's HSD test between mycelia growing without competitors (control) and with other species (bicultures). Adjusted *p* values were calculated with Tukey's HSD test.

Species	Comparison	Estimate	Estimate SE	*z*	Adjusted *p*
*Beauveria felina*	*G. polymorpha* *‐R. mucilaginosa*	−0.117577	0.003403	−34.55	< 2 × 10^−16^
	Control‐ *G. polymorpha*	−0.170697	0.003586	−47.60	< 2 × 10^−16^
	Control‐ *R. mucilaginosa*	−0.053121	0.003527	−15.06	< 2 × 10^−16^
*Gliomastix polymorpha*	*B. felina‐R. mucilaginosa*	0.0230827	0.0033551	6.880	< 10^−5^
	Control‐ *R. mucilaginosa*	0.0240300	0.0033139	7.251	< 10^−5^
	Control‐ *B. felina*	0.0009473	0.0033551	0.282	0.957
*Rhodotorula mucilaginosa*	*B. felina‐G. polymorpha*	−0.120213	0.006555	−18.34	< 2 × 10^−16^
	Control‐ *B. felina*	−0.091859	0.006934	−13.25	< 2 × 10^−16^
	Control‐ *G. polymorpha*	−0.212072	0.006970	−30.43	< 2 × 10^−16^

**FIGURE 3:**
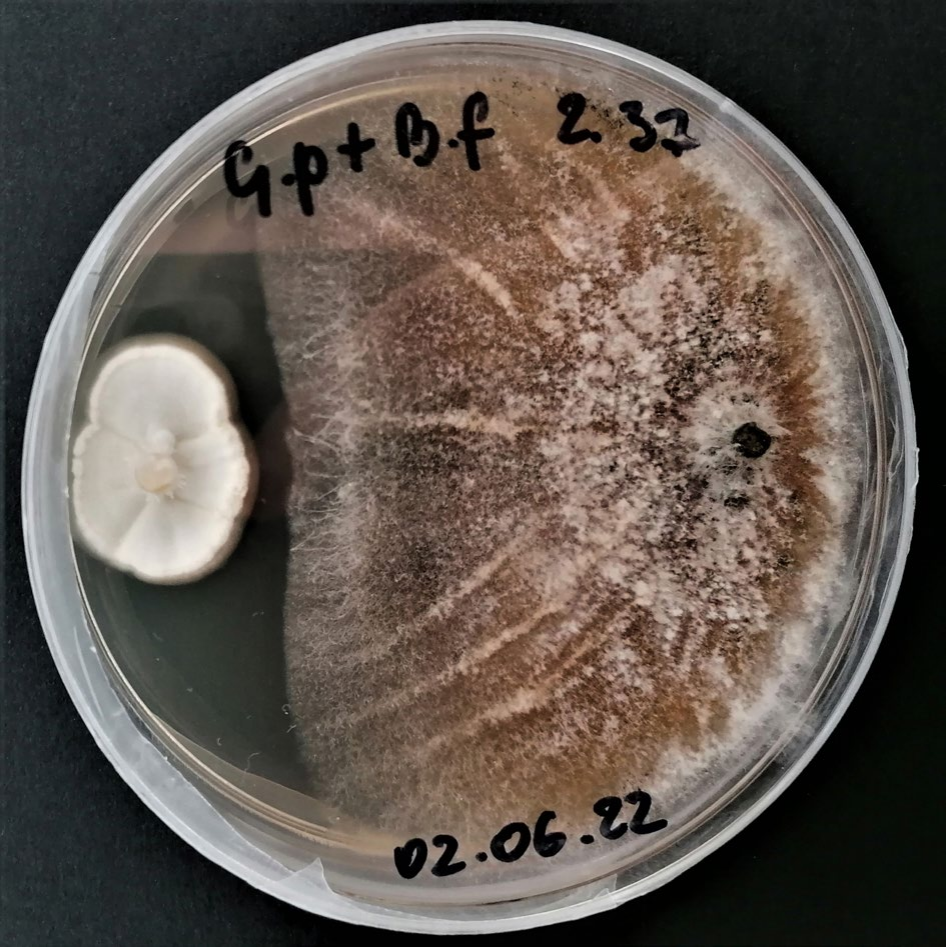
Inhibition zone as a biotic effect of the interaction between *Gliomastix polychrome* and *Beauveria feline* cultured in vitro. Fot. M. Lembicz.

In the case of interaction between *B. felina* and *G. polychroma*, in 26 out of 40 analysed samples (65% of the total), an inhibition zone was formed between their mycelia (Figure 3). Despite the markedly faster growth rate of G. *polychroma* than its competitor, the growth of its fungal network was inhibited near the *B. felina* mycelium.

## 4 Discussion

In this study, we tested the mycelium growth rate of *Beauveria felina*—a candidate for biocontrol of crop pathogens—in the presence of competitors: other endophytic fungi. Our study was based on knowledge about the common occurrence and crucial role of fungal endophytes in the plant world (Saikkonen et al. 2004; Cheplick and Faeth 2009; Gupta et al. 2023). Thus, if a fungus is supposed to be a biocontrol agent, then it is necessary to check how it responds to the presence of other fungal species, forming the fungal microbiome of the plant. There can be several scenarios of such interactions. A fungal biocontrol agent applied to fight a specific crop disease may, for example, inhibit the growth of other fungal species present in the plant, including those that have a positive effect on plant growth and reproduction, as well as defence against other pathogens. Some fungi may inhibit or stimulate the mycelium growth of the biocontrol agent. Consequently, depending on the network effects of fungus–fungus interactions, the effectiveness of the biocontrol agent could be increased or decreased.

In our study, the selected fungal endophytes, cultured with the biocontrol agent, stimulated its growth. This result supports earlier reports that indicate *B. felina* as a good candidate for biocontrol in crops (e.g., Du et al. 2020). In the presence of *B. felina* , no significant change in growth rate occurred.

The interaction between *B. felina* and *G. polychroma* is interesting, as, despite the significant increase in the mycelium growth rate of *B. felina* , no significant change was observed in the growth rate of *G. polychroma*; however, an inhibition zone was formed between their mycelia. This was probably caused by chemical compounds produced by them, which were released into the growth medium and/or into the air (volatile compounds). These metabolites may be inhibitors for hyphal apical cells, which are the beginning of mycelial growth. Which species produce the metabolites that significantly slow down the spread of the mycelium remains unclear. This interaction indicates that *B. felina* has a different effect on the growth rate of other endophytes. While it accelerates the growth of *R. mucilaginosa* , its influence on *G. polychroma* is negligible. However, the presence of *G. polychroma* or *R. mucilaginosa* accelerated the growth of *B. felina* .

An effective biocontrol agent must produce chemical compounds that fight the targeted pathogen. Moreover, the biocontrol agent and its metabolites should not have any negative effect on plant fitness and its mycobiome, which could be transmitted to its future offspring. It is difficult to assess the profits and losses in the interactions to which we introduce an additional fungal species or its metabolites. However, we created a new, artificial symbiosis that began a new evolutionary game. Its effects must be studied before applying the selected biocontrol agent to crop on a large scale. From the evolutionary standpoint, it is difficult to achieve certainty. The measured initial effects of a biocontrol agent may differ from long‐term ones, which may be undesirable.

In conclusion, the introduction of a fungal biocontrol agent to ecosystem services requires research based on a suitable biotechnological strategy as well as studies of its ecological and evolutionary effects on the plant treated with the biocontrol agent because plants are organisms that include more than their own genome. The new concept of holobionts defines the plant as a host coexisting with the microbiota, that is, fungi and bacteria. Plants are also reservoirs and media for the replication of viruses and other infectious particles (Vandenkoornhuyse et al. 2015; Rosenberg and Zilber‐Rosenberg 2016). Thus, the interior of a plant host is a complex, dynamic environment full of interspecific interactions. The introduction of another species—a new partner that should bring desirable effects—requires knowledge about its influence on pre‐existent interactions and relations taking place in the holobiont.

## Author Contributions

**Wojciech Pijanowski:** methodology. **Sebastian Chmielewski:** methodology. **Wojciech Wysoczański:** writing – original draft, conceptualization, writing – review and editing, methodology. **Polina Havrysh:** validation. **Katarzyna Turnau:** conceptualization. **Marlena Lembicz:** writing – original draft, funding acquisition, supervision, writing – review and editing.

## Conflicts of Interest

The authors declare no conflicts of interest.

## Data Availability

The data that supports the findings of this study are available in the manuscript.

## References

[emi470067-bib-0001] Adeleke, B. S. , M. S. Ayilara , S. A. Akinola , and O. O. Babalola . 2022. “Biocontrol Mechanisms of Endophytic Fungi.” Egyptian Journal of Biological Pest Control 32: 46. 10.1186/s41938-022-00547-1.

[emi470067-bib-0002] Agrawal, R. , A. Verma , R. R. Singhania , S. Varjani , C. Di Dong , and A. K. Patel . 2021. “Current Understanding of the Inhibition Factors and Their Mechanism of Action for the Lignocellulosic Biomass Hydrolysis.” Bioresource Technology 332, no. 125: 42. 10.1016/j.biortech.2021.125042 33813178

[emi470067-bib-0003] Alam, B. , J. Lǐ , Q. Gě , et al. 2021. “Endophytic Fungi: From Symbiosis to Secondary Metabolite Communications or Vice Versa?” Frontiers in Plant Science 12: 3060. 10.3389/fpls.2021.791033 PMC8718612 34975976

[emi470067-bib-0004] Backman, P. A. , and R. A. Sikora . 2008. “Endophytes: An Emerging Tool for Biological Control.” Biological Control 46, no. 1: 1–3.

[emi470067-bib-0005] Baiyee, B. , C. Pornsuriya , S. I. Ito , and A. Sunpapao . 2019. “Trichoderma Spirale T76‐1 Displays Biocontrol Activity Against Leaf Spot on Lettuce ( *Lactuca sativa* L.) Caused by *Corynespora cassiicola* or *Curvularia aeria* .” Biological Control 129: 195–200.

[emi470067-bib-0007] Cheplick, G. P. , and S. H. Faeth . 2009. Ecology and Evolution of the Grass‐Endophyte Symbiosis. New York, NY: Oxford University Press, Inc.

[emi470067-bib-0008] Du, F. Y. , X. M. Li , Z. C. Sun , L. H. Meng , and B. G. Wang . 2020. “Secondary Metabolites With Agricultural Antagonistic Potentials From *Beauveria felina*, a Marine‐Derived Entomopathogenic Fungus.” Journal of Agricultural and Food Chemistry 68: 14824–14831. 33322905 10.1021/acs.jafc.0c05696

[emi470067-bib-0009] Gardes, M. , and T. D. Bruns . 1993. “ITS Primers With Enhanced Specificity for Basidiomycetes—Application to the Identification of Mycorrhizae and Rusts.” Molecular Ecology 2: 113–118. 10.1111/j.1365-294X.1993.tb00005.x. 8180733

[emi470067-bib-0010] Green, M. R. , and J. Sambrook . 2017. “Precipitation of DNA With Isopropanol.” Cold Spring Harbor Protocols 8: pdb‐prot093385. 10.1101/pdb.prot093385 28765297

[emi470067-bib-0011] Gupta, A. , V. Meshram , M. Gupta , et al. 2023. “Fungal Endophytes: Microfactories of Novel Bioactive Compounds With Therapeutic Interventions; a Comprehensive Review on the Biotechnological Developments in the Field of Fungal Endophytic Biology Over the Last Decade.” Biomolecules 13, no. 7: 1038. 37509074 10.3390/biom13071038 PMC10377637

[emi470067-bib-0012] Jiang, M. , S. Chen , X. Lu , et al. 2023. “Integrating Genomics and Metabolomics for the Targeted Discovery of New Cyclopeptides With Antifungal Activity From a Marine‐Derived Fungus *Beauveria felina* .” Journal of Agricultural and Food Chemistry 71, no. 25: 9782–9795. 10.1021/acs.jafc.3c02415. 37310400

[emi470067-bib-0013] Kassambara, A. 2023. “rstatix: Pipe‐Friendly Framework for Basic Statistical Tests.” R Package Version 0.7.2.

[emi470067-bib-0014] Latz, M. A. , B. Jensen , D. B. Collinge , and H. J. Jørgensen . 2018. “Endophytic Fungi as Biocontrol Agents: Elucidating Mechanisms in Disease Suppression.” Plant Ecology and Diversity 11, no. 5–6: 555–567.

[emi470067-bib-0015] Menzler‐Hokkanen, I. 2006. “Socioeconomic Significance of Biological Control.” In An Ecological and Societal Approach to Biological Control, 13–25. Dordrecht, the Netherlands: Springer.

[emi470067-bib-0016] Morais‐Urano, R. P. , A. C. Chagas , and R. G. Berlinck . 2012. “Acaricidal Action of Destruxins Produced by a Marine‐Derived *Beauveria felina* on the Bovine Tick Rhipicephalus (Boophilus) Microplus.” Experimental Parasitology 132, no. 3: 362–366. 22955115 10.1016/j.exppara.2012.08.011

[emi470067-bib-0017] Pedras, M. S. C. , L. I. Zaharia , and D. E. Ward . 2002. “The Destruxins: Synthesis, Biosynthesis, Biotransformation and Biological Activity.” Phytochemistry 59: 579–596. 10.1016/S0031-9422(02)00016-X. 11867090

[emi470067-bib-0018] Ramanujam, B. , B. Poornesha , A. Kandan , M. Mohan , and G. Sivakumar . 2021. “Natural Occurrence of Entomopathogenic Fungus *Beauveria felina* (DC.) JW Carmich on Fall Armyworm, *Spodoptera frugiperda* (JE Smith).” Journal of Entomology and Zoology Studies 9, no. 3: 140–143.

[emi470067-bib-0019] Rodriguez, R. J. , J. F. White Jr. , A. E. Arnold , and A. R. A. Redman . 2009. “Fungal Endophytes: Diversity and Functional Roles.” New Phytologist 182, no. 2: 314–330. 10.1111/j.1469-8137.2009.02773.x. 19236579

[emi470067-bib-0020] Rosenberg, E. , and I. Zilber‐Rosenberg . 2016. “Microbes Drive Evolution of Animals and Plants: The Hologenome Concept.” MBio 7, no. 2: e01395. 10.1128/mBio.01395-15. PMC4817260 27034283

[emi470067-bib-0021] Saikkonen, K. , P. E. Gundel , and M. Helander . 2013. “Chemical Ecology Mediated by Fungal Endophytes in Grasses.” Journal of Chemical Ecology 39: 962–968. 10.1007/s10886-013-0310-3. 23797930

[emi470067-bib-0022] Saikkonen, K. , P. Wäli , M. Helander , and S. H. Faeth . 2004. “Evolution of Endophyte–Plant Symbioses.” Trends in Plant Science 9, no. 6: 275–280. 10.1016/j.tplants.2004.04.005. 15165558

[emi470067-bib-0023] Segaran, G. , and M. Sathiavelu . 2019. “Fungal Endophytes: A Potent Biocontrol Agent and a Bioactive Metabolites Reservoir.” Biocatalysis and Agricultural Biotechnology 21, no. 101: 284.

[emi470067-bib-0024] Sirikamonsathien, T. , M. Kenji , and T. Dethoup . 2023. “Potential of Endophytic Trichoderma in Controlling Phytophthora Leaf Fall Disease in Rubber ( *Hevea brasiliensis* ).” Biological Control 179, no. 105: 175.

[emi470067-bib-0025] Sprouffske, K. , and A. Wagner . 2016. “Growthcurver: An R Package for Obtaining Interpretable Metrics From Microbial Growth Curves.” BMC Bioinformatics 17: 1–4. 27094401 10.1186/s12859-016-1016-7 PMC4837600

[emi470067-bib-0026] Thambugala, K. M. , D. A. Daranagama , A. J. L. Phillips , S. D. Kannangara , and I. Promputtha . 2020. “Fungi vs. Fungi in Biocontrol: An Overview of Fungal Antagonists Applied Against Fungal Plant Pathogens.” Frontiers in Cellular and Infection Microbiology 10: 604923. 10.3389/fcimb.2020.604923. 33330142 PMC7734056

[emi470067-bib-0027] Vandenkoornhuyse, P. , A. Quaiser , M. Duhamel , A. Le Van , and A. Dufresne . 2015. “The Importance of the Microbiome of the Plant Holobiont.” New Phytologist 206: 1196–1206. 10.1111/nph.13312. 25655016

[emi470067-bib-0028] Vázquez de Aldana, B. R. , I. Zabalgogeazcoa , R. Rubio de Casas , C. A. García , and B. García Criado . 2010. “Relationships Between the Genetic Distance of *Epichloë festucae* Isolates and the Ergovaline and Peramine Contents of Their *Fesuca rubrahosts* .” Annals of Applied Biology 156: 51–61.

[emi470067-bib-0029] Vázquez, M. J. , M. I. Albarrán , A. Espada , A. Rivera‐Sagredo , E. Díez , and J. A. Hueso‐Rodríguez . 2005. “A New Destruxin as Inhibitor of Vacuolar‐Type H+‐ATPase of *Saccharomyces cerevisiae* .” Chemistry and Biodiversity 2: 123–130. 10.1002/cbdv.200490163. 17191925

[emi470067-bib-0030] Vega, F. E. 2018. “The Use of Fungal Entomopathogens as Endophytes in Biological Control: A Review.” Mycologia 110, no. 1: 4–30. 29863999 10.1080/00275514.2017.1418578

[emi470067-bib-0031] White, J. F., Jr. , and C. W. Bacon . 2012. “Special Issue: The Secret World of Endophytes.” Fungal Ecology 5: 3–288.

[emi470067-bib-0032] White, T. , L. S. Bruns , and J. Taylor . 1990. “Amplification and Direct Sequencing of Fungal Ribosomal RNA Genes for Phylogenetics.” In PCR Protocols: A Guide to Methods and Applications, edited by M. Innis , D. Gelfand , J. Sninsky , and T. White , 315–322. San Diego, CA: Academic Press.

[emi470067-bib-0033] Zhang, Y. , T. Han , Q. Ming , L. Wu , K. Rahman , and L. Qin . 2012. “Alkaloids Produced by Endophytic Fungi: A Review.” Natural Product Communications 7, no. 7: 963–968. 22908594

